# Granulocyte Colony-Stimulating Factor (G-CSF) Protects Oligpdendrocyte and Promotes Hindlimb Functional Recovery after Spinal Cord Injury in Rats

**DOI:** 10.1371/journal.pone.0050391

**Published:** 2012-11-27

**Authors:** Ryo Kadota, Masao Koda, Junko Kawabe, Masayuki Hashimoto, Yutaka Nishio, Chikato Mannoji, Tomohiro Miyashita, Takeo Furuya, Akihiko Okawa, Kazuhisa Takahashi, Masashi Yamazaki

**Affiliations:** 1 Department of Orthopaedic Surgery, Chiba University Graduate School of Medicine, Chuo-Ku, Chiba, Japan; 2 Department of Orthopaedic Surgery, Chiba Aoba Municipal Hospital, Chuo-Ku, Chiba, Japan; 3 Department of Orthopaedic Surgery, Chiba Medical Center, Chuo-Ku, Chiba, Japan; Universidad de Castilla-La Mancha, Spain

## Abstract

**Background:**

Granulocyte colony-stimulating factor (G-CSF) is a protein that stimulates differentiation, proliferation, and survival of cells in the granulocytic lineage. Recently, a neuroprotective effect of G-CSF was reported in a model of cerebral infarction and we previously reported the same effect in studies of murine spinal cord injury (SCI). The aim of the present study was to elucidate the potential therapeutic effect of G-CSF for SCI in rats.

**Methods:**

Adult female Sprague-Dawley rats were used in the present study. Contusive SCI was introduced using the Infinite Horizon Impactor (magnitude: 200 kilodyne). Recombinant human G-CSF (15.0 µg/kg) was administered by tail vein injection at 1 h after surgery and daily the next four days. The vehicle control rats received equal volumes of normal saline at the same time points.

**Results:**

Using a contusive SCI model to examine the neuroprotective potential of G-CSF, we found that G-CSF suppressed the expression of pro-inflammatory cytokine (IL-1 beta and TNF- alpha) in mRNA and protein levels. Histological assessment with luxol fast blue staining revealed that the area of white matter spared in the injured spinal cord was significantly larger in G-CSF-treated rats. Immunohistochemical analysis showed that G-CSF promoted up-regulation of anti-apoptotic protein Bcl-Xl on oligpodendrocytes and suppressed apoptosis of oligodendrocytes after SCI. Moreover, administration of G-CSF promoted better functional recovery of hind limbs.

**Conclusions:**

G-CSF protects oligodendrocyte from SCI-induced cell death via the suppression of inflammatory cytokines and up-regulation of anti-apoptotic protein. As a result, G-CSF attenuates white matter loss and promotes hindlimb functional recovery.

## Introduction

Acute spinal cord injury (SCI) is divided into two pathological phases termed primary and secondary injury [1]. The primary injury consists of focal tissue destruction caused by direct mechanical trauma. This physical insult then initiates the second phase of injury which is a pathophysiological reaction of spinal cord. Apoptosis of neurons and glial cells left intact by the initial trauma occurs during the secondary phase. In addition, oligodendrocytes distant from the immediate site of injury undergo apoptosis. Maximal cell death occurs one week after injury and leads directly to demyelination [2]. Several *in vivo* studies have demonstrated that the amount of spared white matter correlates to residual locomotor function [3], [4]. Thus, protection of oligodendrocytes from apoptotic cell death might reduce demyelination and improve functional recovery. Many factors could exacerbate the secondary phase of injury, including vascular changes, increased concentrations of free radicals and free fatty acids, ionic mechanisms of axonal injury, glutamate excitotoxicity, and immune and inflammatory reactions [5]. Currently, high-dose methylprednisolone (MP) in acute SCI is an accepted treatment for attenuation of secondary injury [6]. However, it has become controversial in recent years due to the risk of serious adverse effects and its modest neurological benefits [7]. Therefore, development of new drug therapies which can substitute for high-dose MP is an area of intense study.

Granulocyte colony-stimulating factor (G-CSF) is a 19.6 kDa glycoprotein that was initially identified as a serum factor that induced differentiation of a murine myelomonocytic leukemic cell line [8]. It is widely known as a hematopoietic cytokine that promotes survival, proliferation and differentiation of cells of the neutrophil lineage [8], [9]. It is used clinically for patients with leukocytopenia and for donors of peripheral blood-derived hematopoietic progenitor cells prior to collection for transplantation [10].

Within the central nervous system (CNS), G-CSF has pleiotropic actions. In recent years, the beneficial effects of G-CSF have been demonstrated in rodent stroke models [11]–[15]. Moreover, clinical trials of G-CSF for stroke reported its safety and feasibility [16]. In the case of SCI, several research groups including us previously reported that G-CSF treatment promoted functional recovery in the mouse and rat SCI models [17]–[22].

Although the beneficial effects of G-CSF on neurons are partially understood, little is known about G-CSF-mediated reduction of apoptosis of oligodendrocytes after SCI. Therefore we hypothesized that G-CSF could attenuate apoptosis of oligodendrocytes and, as a result, improve white matter preservation and functional recovery. This may represent another mechanism by which G-CSF provides neuroprotection following SCI. In the present study, our aim was to assess the anti-apoptotic effects of G-CSF on oligodendrocytes and to elucidate the mechanism using the rat contusive SCI model.

## Results

### G-CSF Receptor (G-CSFR) Expression

**Figure 1:**
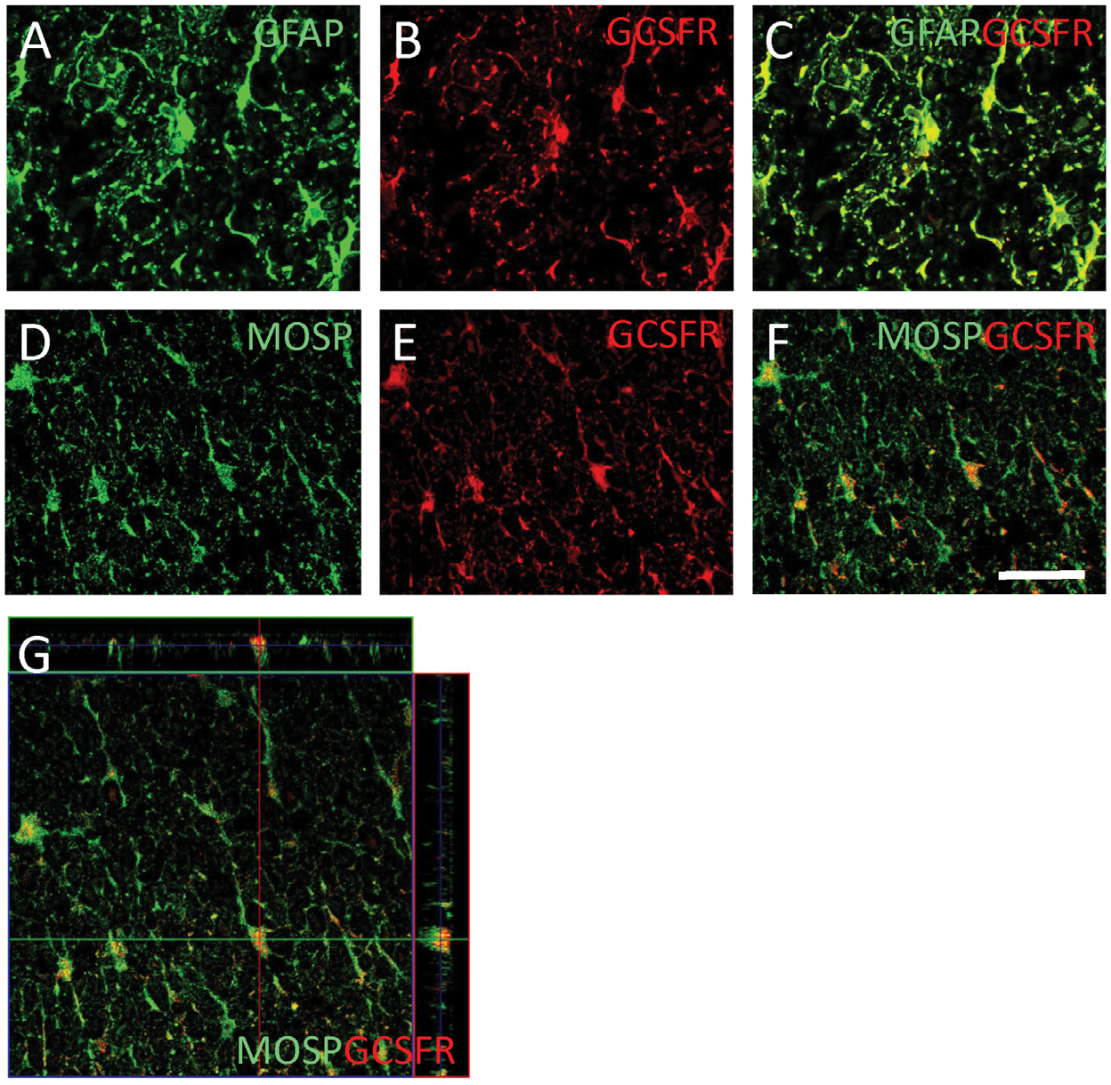
Granulocyte colony-stimulating factor receptor (G-CSFR) expression. Immunofluorescent double labeling for G-CSFR and cell-specific markers 1 week after surgery in vehicle-treated rats. Double-positive cells for G-CSFR and glial fibrillary acidic protein (GFAP, marker for astrocytes; A–C) and G-CSFR and myelin oligodendrocyte specific protein (MOSP, marker for oligodendrocytes; D–G) were detected. To show co-localization precisely, positive signal for G-CSFR/GFAP and G-CSFR/MOSP were detected using confocal laser microscopy (A–F) and 3-dimensional image was reconstructed (G). Bars = 50 µm.

To assess the expression of G-CSFR, we performed immunofluorescence analysis on histological sections of spinal cords. The data revealed that G-CSFR was expressed on neurons, astrocytes and oligodendrocytes in normal spinal cord (Fig. S1A–C). One week after injury, G-CSFR was expressed on glial fibrillary acidic protein (GFAP) -positive astrocytes and myelin oligodendrocyte-specific protein (MOSP) -positive oligodendrocytes (Fig. 1). Quantification of G-CSFR/MOSP double-positive cells showed significant increase of the number of MOSP-positive oligpodendrocytes in the G-CSF-treated group 2 mm rostral and caudal to the epicentre (Fig. S1D). Almost all of MOSP-positive oligodendrocytes expressed G-CSFR in both the vehicle and G-CSF groups (Fig. S1D).

### Expression of Inflammatory Cytokines after SCI

**Figure 2:**
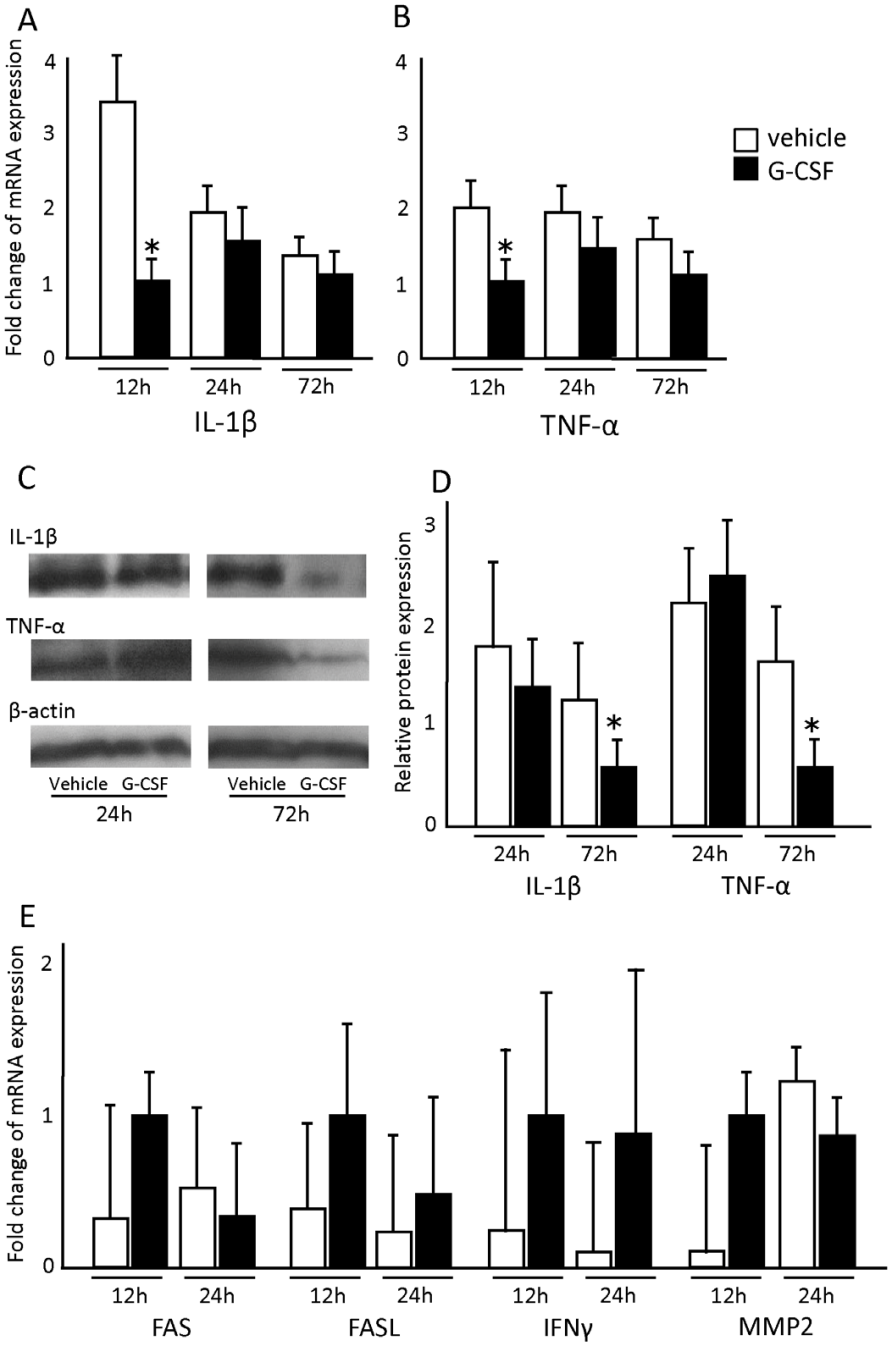
G-CSF suppressed pro-inflammatory cytokine expression. Real time quantitative PCR and western blot analysis were performed to quantify mRNA and protein expression of pro-inflammatory cytokines in the acute phase of spinal cord injury. Values obtained were normalized to value of 18S ribosomal RNA expression and were expressed as the fold-increase over values of the G-CSF group 12 hours after surgery. The expression of interleukin-1 beta (IL-1 β, A) and tumor necrosis factor-alpha (TNF-α, B) mRNA was significantly suppressed in the G-CSF group (closed column) compared with the vehicle group (open column) 12 hours after surgery. Western blot analysis revealed that G-CSF suppressed the expression level of IL-1 β (C, upper row right, D, closed column, p<0.05) and TNF-α (C, middle row right, D, closed column, p<0.05). There was no significant difference between the vehicle and G-CSF groups in mRNA expression of FAS, FASL, IFN-γ, MMP-2 and MMP-9. Values are mean±SEM. *p<0.05.

To detect the anti-inflammatory effects of G-CSF in the SCI model, we performed Real-Time PCR for interleukin 1-beta (IL-1β), tumor necrosis factor-alpha (TNF-α), FAS, FAS ligand (FASL), interferon-gamma (IFN-γ), matrix metalloproteinase-2 (MMP2) and matrix metalloproteinase-9 (MMP9) (Fig. 2). The study revealed that 12 h after surgery, expression of IL-1β and TNF-α mRNAs was significantly suppressed in the G-CSF group (Fig. 2 A, B, closed columns) compared to the vehicle control group (Fig. 2 A, B, open columns). Specifically, expression of IL-1β mRNA in the G-CSF group was 3.36-fold lower than in the vehicle group (Fig. 2 A, p<0.05). For TNF-α mRNA in the G-CSF group, expression was 1.98-fold lower than in the vehicle group (Fig. 2 B, p<0.05). Twenty-four h and 72 h following surgery, expression of IL-1β and TNF-α mRNAs tended to be lower than controls; however, the differences were not statistically significant. The results of Real-Time PCR for the other factors showed no significant difference between the vehicle and G-CSF-treated groups.

To further confirm G-CSF-mediated attenuation of SCI-induced up-regulation of IL-1β and TNF-α, we performed western blot analysis for IL-1β and TNF-α on protein samples extracted from spinal cord with or without G-CSF treatment 24 and 72 h following spinal cord injury. Western blot analysis revealed that G-CSF suppressed protein expression of IL-1β and TNF-α 72 hours after the injury (Fig. 2C, D, p<0.05).

### Enumeration of Early Inflammatory Leukocytes and Microglia/macrophages

**Figure 3:**
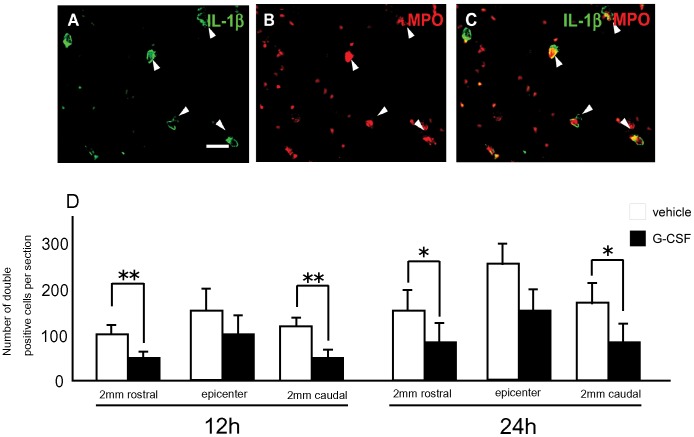
G-CSF decreased the number of IL- β expressing leukocytes. Immunohistochemistry for IL-1β and myeloperoxidase (MPO) in the acute phase of injury. Near the lesion epicenter, IL-1β-positive round cells were also positive for MPO, a marker for leukocytes (A–C, arrowheads). The number of double positive cells for IL-1β and MPO was significantly smaller in the G-CSF group (D, closed column) than in the vehicle group (D, open column). Bars = 50 µm Values are mean±SEM. *p<0.05.

Immunohistochemistry for IL-1β and myeloperoxidase (MPO, a marker for leukocyte) revealed that the frequencies of cells positive for MPO were not statistically different in the two groups 12 and 24 h after surgery (data not shown). In contrast, the frequencies of cells positive for both IL-1β and MPO was significantly smaller in the G-CSF group than in the vehicle group 12 and 24 h after surgery (Fig. 3D). Twelve hours after surgery, the mean numbers of double positive cells per section in the G-CSF group were 52±3.8 in 2 mm rostral to the epicenter and 56±2.5 in 2 mm caudal to the epicenter, respectively (Fig. 3D). In contrast, the frequencies of double positive cells in the vehicle group were considerably higher 12 hours after surgery (103±4.0 per section in 2 mm rostral to the epicenter and 118±5.3 per section in 2 mm caudal to the epicenter, respectively; Fig. 3D). Twenty-four hours after the injury, the G-CSF group showed decrease of the number of IL-1β and MPO double-positive cells as same as 12 hours after injury (Fig. 3D). Double immunofluorescence study for ionized calcium-binding adaptor molecule 1 (Iba-1, as a marker for activated microglia and macrophages) and inducible nitric oxide synthase (iNOS, as a marker for Th1-driven activation of microglia/macrophages) or arginase-1 (a marker for Th2-driven activation of microglia/macrophages) was performed to elucidate G-CSF-mediated reaction and phenotypic alteration of macrophage/microglia. The number of Iba-1-positive cells in the G-CSF group was significantly smaller than that in the vehicle group in the rostral and caudal segments (Fig. S3), whereas the ratios between iNOS and arginase-1 did not change in both the vehicle and G-CSF groups in lesioned spinal cord at any segments observed (Fig. S3).

### Suppressed Apoptosis of Oligodendrocytes

**Figure 4:**
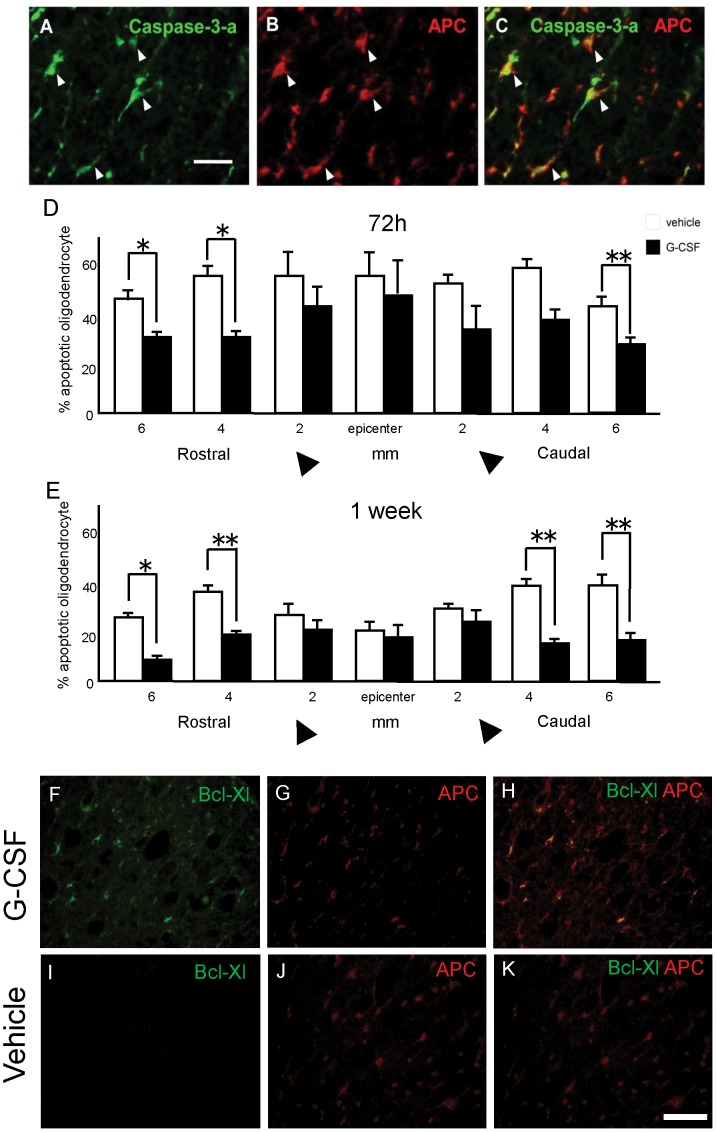
G-CSF suppressed apoptosis of oligodendrocytes *and promoted anti-apoptotic protein Bcl-Xl on oligodendrocytes.* Immunohistochemistry for adenomatous polyposis coli (APC; a marker for oligodendrocytes) and activated caspase 3 (a marker for apoptotic cells) in the acute phase of spinal cord injury. Representative figure of cells double-positive for APC and activated caspase 3 in the vehicle group 4 mm rostral to the lesion epicenter 1 week after injury is shown (A–C, arrowheads). The percentage of apoptotic oligoendrocytes was significantly smaller in the G-CSF group (closed column) than that in the vehicle group (open column) 72 hours (D) and one week (E) after surgery. *In vehice-treated control rats, Bcl-Xl/APC doubler-positive cells were not detected (I–K). In contrast, Bcl-Xl/APC doubler-positive Bcl-Xl expressing oligodendrocytes were observed in the spinal cord of G-CSF-treated rats (F–H).* Bars = 50 µm (A–C) and 100 µm (F–K). Values are mean±SEM. *p<0.05, **p<0.01.

Near the epicenter of the injury, apoptotic oligodendrocytes (adenomatous polyposis coli; APC^+^ and caspase 3^+^ cells) were observed (Fig. 4 A–C, arrowheads). The percentages of apoptotic oligodendrocytes were significantly smaller in the G-CSF group than that in the vehicle group both 72 h and 1week after surgery. Specifically, 72 hours after surgery, the mean percentages of apoptotic oligodendrocytes among the sections were 32.0% in the G-CSF group and 47.8% in the vehicle group (Fig. 4 D). The percentages of apoptotic oligodendrocytes were significantly suppressed in most sections: 4 mm and 6 mm rostral to the epicenter (p<0.05) and 6 mm caudal to the epicenter (p<0.01). Significance was not reached for the section 4 mm caudal to the epicenter. One week after surgery, the mean percentages of apoptotic oligodendrocytes among sections were 13.9% in the G-CSF group and 35.2% in the vehicle group (Fig. 4 E). The percentages of apoptotic oligodendrocytes were significantly suppressed in all of the sections: 6 mm rostral to the epicenter (p<0.05), and 4 mm rostral and 4 mm and 6 mm caudal to the epicenter (p<0.01). To further confirm the results of immunohistochemistry for apoptotic oligodendriocytes, we performed double immunofluorescence study for MOSP as another marker for oligodendrocytes and activated caspase-3 as a marker for apoptotic cells (Fig. S4). The staining pattern was similar to that of the double fluorescence study for APC and activated caspase-3, suggesting that the data of apoptotic oligodendrocytes were convincing.

There was no APC- and Bcl-Xl-double positive cells in vehicle control rats 1 week after injury (Fig. 4 I–K), whereas a part of APC-positive cells simultaneously expressed Bcl-Xl in G-CSF-treated rat 1 week after injury (average 31.8% of APC-positive cells expressed Bcl-Xl, Fig. 4F–H).

The number of MAP-2-positive neurons was significantly larger in the G-CSF group than that in the vehicle group in the rostral and caudal segments to the lesion epicenter (Fig. S2).

### White Matter Sparing after SCI

**Figure 5:**
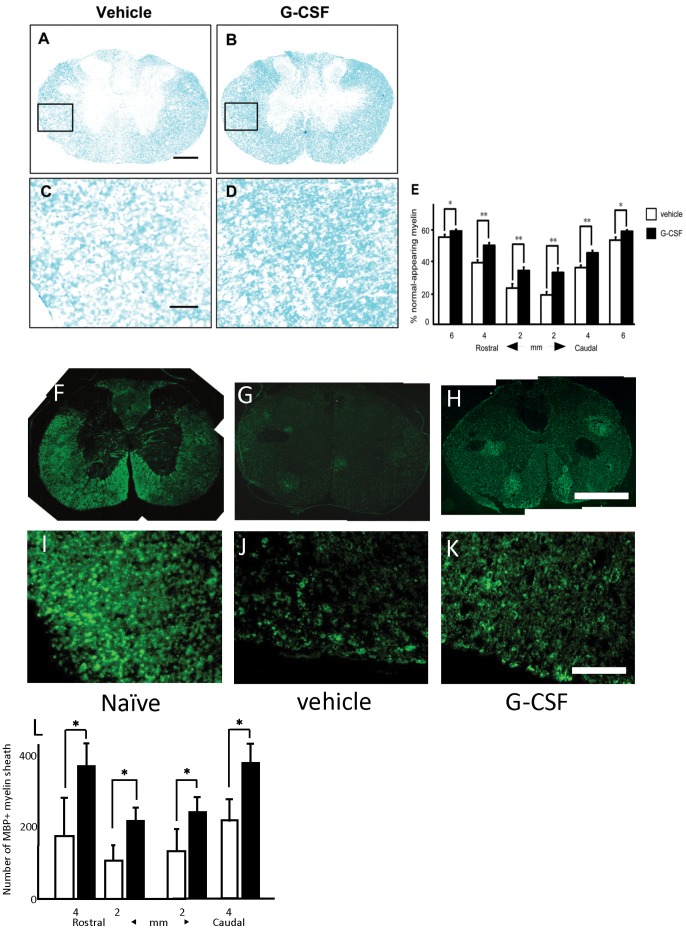
G-CSF attenuated degeneration of white matter myelin. Luxol Fast Blue (LFB) staining and immunofluoresent staining for myelin basic protein (MBP) were performed to quantify spared myelin in the chronic phase of SCI. Figures show LFB staining of the spinal cord 4 mm caudal to the epicenter. Higher magnification revealed better myelin integrity in rats from the G-CSF group (D) than the vehicle group (C). The extent of loss of myelin in the chronic phase of injury (six weeks after surgery) was analyzed (Fig. 4–E). Gray matter of each sample was subtracted from the whole area of the slice to reveal the area of white matter. The actual area of normal-appearing myelin was divided by the white matter to determine the percentage of normal-appearing myelin. The percentage of normal appearing myelin was significantly larger in the G-CSF group (E; closed column) than the vehicle group (E; open column). Immunofluorescence showed that the number of MBP-positive myelin sheath was larger in the G-CSF group than that in the vehicle group (L). Bars = 1 mm (F–H), 500 µm (A, B) and 100 µm (C, D, I–K), values are mean±SEM. *p<0.05, **p<0.01. Values are mean±SEM. *p<0.05.

Luxol fast blue (LFB) staining 6 weeks after injury revealed better myelin integrity and preservation in G-CSF-administered rats in increased magnification of the section (Fig. 5C, D, 4 mm caudal to epicenter). The percentage of normal-appearing myelin in the G-CSF group (Fig. 5E, closed columns) was significantly higher than that in the vehicle group (Fig. 5E, open columns) in all the segments analyzed except for the lesion epicenter.

Immunohistochemistry for myelin basic protein (MBP) 6 weeks after injury showed preservation of myelin sheath in G-CSF-treated animals. The number of MBP-positive myelin sheath in the G-CSF group (Fig. 5H, K and Fig. 5L, closed columns) was significantly larger than that in the vehicle group (Fig. 5G, J and Fig. 5L, open columns).

### Recovery of Hindlimb Motor Function

**Figure 6:**
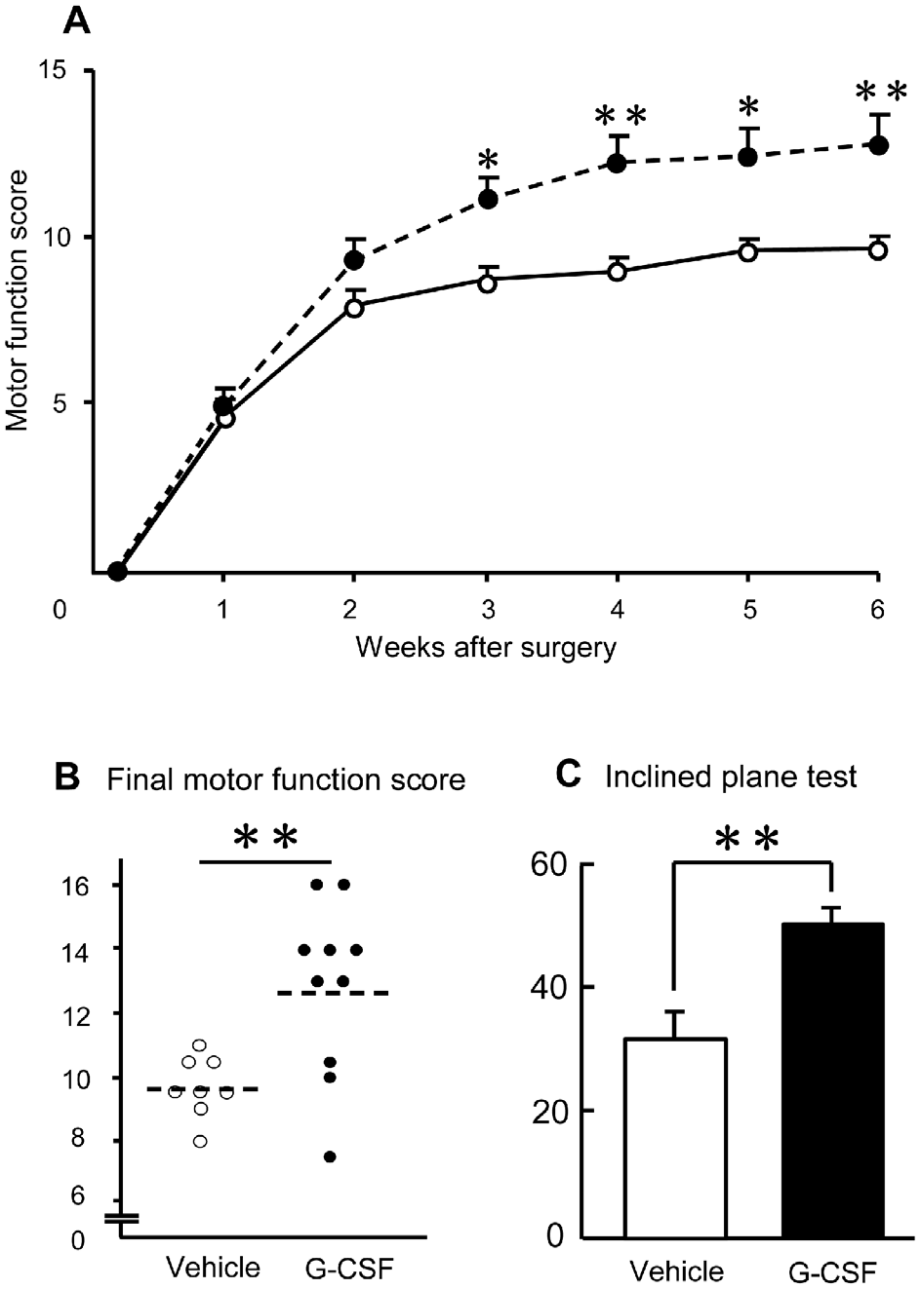
Locomotor recovery. Assessment of hind limb function with the Basso, Beattie and Bresnahan (BBB) locomotor scale (A, B). Time course of recovery of hindlimb function (A) and comparison of final motor function scores (B). Rats from the G-CSF group (closed circle) showed significant recovery compared to rats from the vehicle group (open circle) (A; Repeated measures ANOVA, p<0.05). The final motor function score six weeks after surgery in the G-CSF group (average 12.8; B, closed circle) was significantly higher than that in the vehicle group (average 9.7; B, open circle, p<0.01). The average score in the inclined plane test ten weeks after surgery in the G-CSF group (average 50 degrees; C, closed column) was significantly higher than that in the vehicle group (average 31.7 degrees; C, open column, p<0.01). Values are mean±S.E.M. *p<0.05, **p<0.01.

All rats had a full score (21 points) prior to surgery, and the score dropped to zero immediately after SCI. Hindlimb function showed a significant recovery in rats in the G-CSF group six weeks after surgery compared with that of the vehicle group (Fig. 6A). The average recovery score six weeks post-surgery was significantly higher in the G-CSF group (Fig. 6B, closed circle, p<0.01) than the vehicle group (Fig. 6B, open circle). The average final score in the G-CSF group was 12.5±0.9 (9 to 16), indicating frequent (51–94%) to consistent weight-supported plantar steps *and* occasional (<50%) forelimb-hindlimb coordination. In contrast, the score was 9.5±0.3 (7 to 13) in the control group, indicating plantar placement of the paw with weight support in stance only (i.e., when stationary) or occasional, frequent, or consistent (95–100%) weight-supported dorsal stepping and no plantar stepping. The mean value of the inclined plane test was significantly higher in the G-CSF group than in the vehicle group (50.0 to 31.7, Fig. 6C, p<0.01). Finally, there was good correlation between the percentage of normal myelin and the final motor function score (r = 0.676, p<0.01, not shown). Those results showed that five days treatment with G-CSF had an impact on the animal status six weeks later.

## Discussion

The present results showed the beneficial effects of G-CSF in the setting of SCI. G-CSF significantly reduced injury-induced up-regulation of IL1β and TNF-α expression in mRNA and protein levels and the number of infiltrating leukocytes and activated microglia/macrophages which potentially involve in SCI-induced inflammatory reactions. Furthermore, early treatment with G-CSF significantly attenuated subsequent apoptosis of oligodendrocytes and white matter degeneration, and promoted better long-term functional recovery of the hindlimbs.

It is believed that the injury-induced up-regulation of inflammatory cytokines substantially contributes to secondary injury following SCI. Exogenous administration of IL1β has been shown to exacerbate ischemic damage, whereas administration of an endogenous IL-1β receptor antagonist [23] or a neutralizing antibody [24] reduced brain damage and edema when administered before a stroke in rats. Previous reports showed that G-CSF mediates anti-inflammatory effects after a variety of infections [25]. G-CSF decreases monocytic production of pro-inflammatory cytokines such as IL-1β and TNF-α *in vitro* [25], [26]. And, *in vivo* studies showed that G-CSF also suppresses the expression of IL-1β in a cerebral infarct model [11] and TNF-α in experimental encephalomyelitis [24]. In the current study, G-CSF significantly reduced the expression of IL-1β and TNF-α mRNA in the acute phase after SCI. Moreover, results of immunofluorescence double staining for IL-1β and MPO suggest that G-CSF reduced inflammatory cytokine expression by neutrophils, whereas G-CSF had no influence on the extent of neutrophil infiltration. The discrepancy between the results of real-time PCR and immunofluorescence may be caused by the time lag between transcription and translation. The results obtained here conflict with a report that G-CSF reduced neutrophil infiltration in a model of splanchnic ischemia and reperfusion [27]. This discrepancy might be caused by general differences between the experimental models and/or unique microenvironments within the organs. Furthermore, the present results showed that GCSF decreased the number of activated microglia/macrophages. Microglia/macrophages can involve inflammatory reaction in injured spinal cord. Therefore this data might suggest the other possible anti-inflammatory action of G-CSF.

G-CSF has direct neuroprotective effects against glutamate-induced neuronal death and stroke [11]–[15], [28]. G-CSF elicits its anti-apoptotic effects on neurons via activation of proteins of the STAT family or the PI3-K/Akt pathway, similar to anti-apoptotic effects on neutrophils [13]. In this study of spinal cord injury in the rat, G-CSFR was expressed in neurons, astrocytes and oligodendrocytes, but not in microglia. SCI promoted up-regulation of G-CSFR expression in astrocytes and oligodendrocytes. Moreover, G-CSF treatment did not alter the expression pattern of G-CSFR. Thus, the reduction of oligodendrocyte apoptosis by G-CSF was possibly achieved by several mechanisms as followings. Firstly, G-CSF-mediated suppression of inflammatory cytokine expression might attenuate apoptosis of oligodendrocytes because these inflammatory cytokines are potentially toxic for oligodendrocytes [29], [30]. Secondly, G-CSF-mediated up-regulation of anti-apoptotic proteins in oligodendrocytes might suppress apoptosis. The present results of immunofluorescence double labelling for APC and Bcl-Xl revealed G-CSF-mediated up-regulation of Bcl-Xl in oligodendrocytes. This data is in line with the G-CSF-mediated up-regulation of Bcl-Xl gene expression in spinal cord partial transection model [21]. Finally, G-CSF-mediated attenuation of neuronal death, which action we previously reported [19], and/or promotion of neurite outgrowth [21] could enhance survival of oligodendrocytes because the survival of oligodendrocytes depends on stimuli from axons [31]. The present data of G-CSF-mediated attenuation of loss of MAP-2- positive neurons might support that hypothesis.

The present results showed that G-CSF suppresses apoptosis of oligodendrocytes and white matter degeneration after SCI. There was a direct correlation between the area of spared myelin and final motor function score. This result is in line with previous reports [32], [33] and explains how G-CSF promotes functional recovery. In the murine SCI model, we previously reported that G-CSF attenuates apoptosis of neurons via the activation of signaling pathways downstream from the G-CSFR [19]. Taken together, G-CSF exerts neuroprotective effects for neurons and oligodendrocytes via anti-inflammatory and anti-apoptotic actions, resulting in reduced white matter degeneration and promotion of functional recovery. Other than the anti-inflammatory or anti-apoptotic effects on the CNS, G-CSF can promote mobilization of bone marrow-derived stem cells and their migration into injured spinal cord tissues and promote functional recovery [18], [20]. Furthermore, G-CSF has several actions on the vascular system. For example, G-CSF suppresses brain edema formation after stroke [11] and promotes angiogenesis in stroke [28] and SCI [17] models. Finally, G-CSF stimulates neurogenesis both directly [13] or via the up-regulation of vascular endothelial growth factor [34] All of those mechanisms could potentially contribute to the neuroprotective effects of G-CSF following SCI.

In terms of safety and efficacy, G-CSF has an excellent record in clinical use. In the case of stroke, STAIR presents criteria for drug development [35] and G-CSF fulfills those criteria well. Briefly, those criteria include the following: i) blood-brain barrier penetration, ii) neuroprotective activity in different stroke models including permanent ischemia demonstrated by independent groups, iii) activity shown in different species, iv) well-known pharmacokinetics, and v) functional outcome data. In this context, G-CSF may be a candidate for clinical application in the setting of acute SCI. In that regard, the therapeutic time window is important. In the rat photothrombotic stroke model, G-CSF administrated 72 h after induction of ischemia for ten days improved recovery [36]. G-CSF administration might be effective if delayed even further. Additional studies are needed to define the therapeutic time window of G-CSF treatment for SCI. G-CSF is an attractive candidate for treatment of acute SCI. While greater understanding of the optimal dosage, therapeutic time window and precise mechanism of action is needed, the present results are encouraging. The feasibility of conducting clinical trials of G-CSF treatment for acute SCI patients should be considered.

### Conclusions

G-CSF protects oligodendrocyte from SCI-induced cell death via the suppression of inflammatory cytokines and up-regulation of anti-apoptotic protein. As a result, G-CSF attenuates white matter loss and promotes hindlimb functional recovery.

## Materials and Methods

### Animals

All animals were treated and cared for in accordance with the Chiba University School of Medicine guidelines pertaining to the treatment of experimental animals. The present study was approved by Animal Care and Use committee of Chiba University Graduate School of Medicine (the approval number was 20060017). Ninety adult female Sprague-Dawley rats (10–12 weeks old; weight 200–240 g; Japan SLC, Inc. Hamamatsu, Japan) were housed in individual cages and given food and water *ad lib*. Rats were anesthetized with 1.5% halothane in 0.5 L/min oxygen and contused on T9 spinal cord exposed by a T8/9 laminectomy. Contusive SCI was introduced using the Infinite Horizon Impactor (Precision Systems and Instrumentation, Lexington, KY, magnitude: 200 kilo dyne). Upon awakening, rats were evaluated neurologically and were monitored for food and water uptake and urine output. As prophylactic, Bactramin (Chugai, Japan) was added to drinking water.

Rats were randomly assigned to two groups. G-CSF group received recombinant human G-CSF (15.0 µg/kg; kindly provided by Kirin Pharma Co. Ltd., Tokyo, Japan), which was dissolved in normal saline. Vehicle group rats received equal volumes of normal saline at the same time points. All compounds were administered by tail vein injection at 1 h after surgery and daily the next four days. We followed the regimen of drug administration previously reported in the rat brain infarction model [13]. In addition, we performed preliminary experiments in other dose regimen (5, 15, 50 µg/kg/d for 5 days after injury). Those preliminary data suggested that 15 µg/kg/d G-CSF exerts most strong effects. Thus we employed 15 µg/kg/d dose regimen. We collected peripheral blood 1 day after injury and counted leukocyte number, the average of which was 3800 in the vehicle control group and 9700 in the G-CSF group.

### Real-Time Polymerase Chain Reaction (PCR) (Relative Quantitation)

We performed quantitative real-time PCR to determine expression of inflammatory cytokines. First-strand cDNA synthesis and Real-Time PCR were performed as previously described [11]. Briely, for gene analysis, animals of each group were euthanized under pentobarbital anesthesia 12, 24 h and 72h after surgery (n = 5/group) and 10 mm of spinal cord segment including lesion were removed, snap-frozen in liquid nitrogen, and stored at −80°C until use. Total RNA was isolated from spinal cord samples using the RNeasy RNA isolation system (Qiagen Inc.) according to the manufacturer's instructions. Taqman technology (Model 7500 sequence detector, Applied Biosystems, Warrington, UK) was used for quantitative real-time PCR. All samples were run in duplicate. TaqMan probes (labeled with the fluorescent reporter FAM) for interleukin-1 beta (IL-1β), tumor necrosis factor-alpha (TNF-α), FAS, FAS ligand, interleukin-6, interferon-gamma (IFN-γ), matrix metalloproteinase-2 (MMP-2) and matrix metalloproteinase-9 (MMP-9) were designed by Applied Biosystems. Samples were tested in duplicate, and the average values of the threshold cycle (Ct) were used for quantification. To quantify the relative expression of each specific gene, the Ct values were normalized for endogenous 18S ribosomal RNA, and compared with a calibrator using the ΔΔCt method (Ct = Ct_Sample_–Ct_Calibrator_) and converted to a logarithmic value. As calibrator, we used expression in the G-CSF group 12 h after surgery because the expression level of those cytokines in the normal spinal cord was very low. The mean result was further subjected to statistical analysis and expressed as fold–increase.

### Western Blot Analysis

Injured spinal cords (10-mm segments) were homogenized in a homogenization buffer (50 mM Tris-HCl (pH 7.4), 150 mM NaCl, 1% Triton X-100) containing a protease inhibitor cocktail (complete, Roche Diagnostics, Basel, Switzerland). Homogenates were cleared by centrifugation at 14,000 rpm for 10 min at 4°C. Protein concentration of supernatants was measured with Bio-Rad Dc Protein Assay Reagents (Bio-Rad Laboratories, Hercules, CA, USA), and the protein concentration was adjusted to 1 mg/mL by diluting supernatants with a homogenization buffer. Protein samples were mixed with an equal volume of a 2×sample buffer (250 mM Tris-HCl, 4% sodium dodecyl sulfate (SDS), 20% glycerol, 0.02% bromophenol blue, and 10% b-mercaptoethanol). After boiling for 5 min, equal volumes of samples were subjected to 10% SDS-polyacrylamide gel electrophoresis (SDS-PAGE) under reducing conditions, and the proteins were transferred to a polyvinylidene difluoride membrane (Immobilon-P; Millipore Corporation, Billerica, MA, USA). After blocking of the membrane with PBS containing 5% skim milk and 0.05% Tween 20, the membrane was reacted with an anti- IL-1β (BD Biosciences, Franklin lakes, NJ), an anti- TNF-α (BD Biosciences) and an anti-β-actin antibody as a loading control (Santa Cruz Biotechnology, Santa Cruz, CA) antibody. For detection, a horse radish peroxidase-conjugated secondary antibody (Cell Signaling Technology, Beverly, MA, USA) and an ECL chemiluminescence system (GE Healthcare, Piscataway, NJ, USA) were used. Quantification of protein bands was performed using image J software.

### Tissue Preparation

For histological evaluation, animals were perfused transcardially with 4% paraformaldehyde in PBS (pH 7.4) under pentobarbital anesthesia 12, 24, 72 h and one week after surgery (n = 4/group) and six weeks after surgery (n = 10/group). Spinal cord tissue blocks including the lesion epicenter were removed and postfixed in the same fixative overnight, stored in 20% sucrose in PBS at 4°C, and embedded in OCT compound (Sakura Finetechnical, Tokyo, Japan). The cryoprotected samples were frozen and kept at −80°C until use. The samples were cut into serial 10 µm transverse sections with a cryostat and mounted on aminosilane-coated slides (Matsunami, Tokyo, Japan).

### Immunofluorescent Labeling

For immunofluorescent labeling, sections were permeated with 0.3% Triton X and treated for 1 h in blocking solution containing 1% bovine serum albumin and Block Ace (Dainippon Pharma, Japan). Sections were then incubated with the following primary antibodies as indicated: mouse monoclonal anti-G-CSF receptor antibody (G-CSFR, 1∶200, Abcam, Cambridge, UK), rabbit polyclonal anti-microtubule associated protein-2 (MAP-2) antibody (MAP-2, 1∶400, Chemicon Inc, Temecula, CA) for neurons, rabbit polyclonal anti-glial fibrillary acidic protein antibody (GFAP, 1∶400, Sigma, St Louis, MO) for astrocytes, mouse monoclonal anti-adenomatous polyposis coli antibody (APC, 1∶800, Calbiochem, San Diego, CA) or mouse monoclonal anti-myelin oligodendrocyte specific protein IgM antibody (MOSP, 1∶200, Chemicon Inc) for oligodendrocytes, goat polyclonal anti-ionized calcium-binding adaptor molecule 1 (Iba-1,1∶500, Abcam) for microglia, rabbit polyclonal anti-activated caspase 3 antibody (Caspase 3-a, 1∶400, R & D systems, Minneapolis, MN) for apoptotic cells, rabbit polyclonal anti-Bcl-Xl antibody (1∶200, AbD Serotec, Kidlington, UK), mouse monoclonal anti-IL-1β antibody (IL-1β, 1∶200, AbD Serotec), rabbit polyclonal anti-myeloperoxidase antibody (MPO, prediluted, Abcam, Cambridge, UK) for infiltrating inflammatory leukocytes, mouse monoclonal anti-inducible nitric oxide synthase antibody (iNOS, 1∶100, Chemicon Inc.), mouse monoclonal anti-arginase 1 antibody (1∶100, Chemicon Inc.) and mouse anti-myelin basic protein antibody (MBP, 1∶100, Chemicon Inc.) for myelin sheath. The sections were reacted with primary antibodies overnight at 4°C. After incubation with the primary antibodies, sections were washed in PBS and then incubated with secondary antibodies at room temperature for 1 h: Alexa 488- or 594- labeled anti-mouse, anti-rabbit IgG, anti-goat IgG or anti-mouse IgM antibodies (1∶800, Molecular Probes, Eugene, OR). Finally, sections were washed twice in PBS and coverslips were added. The positive signals were observed by fluorescence microscopy (ECLIPSE E600; Nikon, Tokyo, Japan). In case of double staining for G-CSFR/GFAP and G-CSFR/MOSP, the positive signals were detected using confocal laser scanning microscopy (LSM5 PASCAL; Carl Zeiss, Germany). The specificity of the staining procedures was controlled by omitting primary or secondary antibodies.

On the sections from normal spinal cord and one week after surgery, immunofluorescence double labeling for G-CSFR and cell specific markers (MAP-2, GFAP, MOSP and Iba-1) was performed to elucidate the expression of G-CSFR. To detect the influence of G-CSF on G-CSFR expression in oligodendrocytes, quantification was performed by cell count of G-CSFR and MOSP-double positive cells one week after SCI.

To observe the effects of G-CSF on inflammatory cells, immunofluorescence for neutorphils and microglia were performed. Twelve and 24 h after surgery, IL-1β-expressing inflammatory leukocytes were counted as IL-1β- and MPO-double positive cells. To quantitatively analyze the number of IL-1β- and MPO-double positive cells sections were picked from lesion epicenter and 2 mm rostral and caudal segments to the epicenter. Three samples from each segment were observed and a mean value of results was analyzed. The mean values of number of double-positive cells per section were compared between the groups. One week after the injury, phenotype of microglia/macrophage was determined by immunofluorescent double staining for Iba-1/iNOS or Iba-1/arginase-1. The number of total Iba-1-positive cells was counted, then the ratio between iNOS-positivity and arginase-positivity was caluculated.

Apoptotic oligodendrocytes were counted as activated caspase 3 and APC-double positive cells in samples collected 72 h and one week after surgery. For the quantitative analysis of the number of apoptotic oligodendrocytes, sections were picked from epicenter, segments 2 mm, 4 mm and 6 mm rostral and caudal to the epicenter. The percentage of apoptotic oligodendrocyte was calculated by dividing the number of activated caspase 3 and APC-double positive cells by total APC-positive cell number. We also preformed immunefluorescence double labelling for APC and one of the anti-apoptotic protein Bcl-Xl on spinal cord histological sections one week after injury.

To determine the influence of neuronal death on oligodendrocyte survival or death, we performed immunofluorescence for MAP-2 one week after the injury and counted the number of MAP-2-positive neurons in both the vehicle and G-CSF-treated rats.

For quantification of immunofluorescence data, every fifth ten-µm transverse section (50 µm apart) was picked from epicenter, 2 mm, 4 mm and 6 mm rostral or caudal to the lesion epicenter. At least ten sections per each animal were counted, resulting in coverage for 500 µm area of spinal cord in each segment. To count positive cells, we used Scion Image computer analysis software (version beta 4.0.3, Scion Corporation, Frederick, MA).

### Myelin Sparing and Oligodendrocyte Cell Count

We performed luxol fast blue (LFB) staining and immunofluorescence for MBP to measure an area of spared white matter myelin six weeks after surgery. The LFB-positive area and total area of white matter of the same section was calculated using Scion Image computer analysis software. Gray matter was subtracted from the whole area of the slice to reveal the area of white matter. To determine the percentage of normal-appearing myelin, the area of the LFB stain was divided by the area of the white matter (Fig. 5 E). Immunofluorescent labeling for MBP was performed to quantify the number of MBP-positive myelin sheath six weeks after surgery as described above. The number of MBP-positive myelin sheath in white matter was counted (Fig. 5 F). Quantification was done as described above.

### Assessment of Locomotor Activity

The recovery of rat hindlimb function in both groups (n = 10 in the G-CSF group and n = 8 in the vehicle group) was determined by measuring the hindlimb motor function score with Basso, Beattie and Bresnahan locomotor scale (BBB scale [37]). Rats were allowed to move freely on the open field with a rough surface for five min at each time tested. The hindlimb movement of rats was scored by two independent observers who were unaware of the treatment. Measurement of motor function was performed weekly following the sixth week after surgery.

In another subset of rats treated the same as above, the inclined plane test was performed ten weeks after surgery as previously described [38] (n = 5 in each group). The highest degree of inclination was defined as that at which the animal could maintain its position for five seconds on two separate trials.

### Statistical Analysis

Results of immunohistochemical studies, percentages of normal-appearing myelin, final motor function scores and inclined plane tests were subjected to Student's t-test. Recovery of motor function scores was subjected to Repeated Measures ANOVA followed by post hoc test using Fisher's Protected Least Signicicant Difference test. Percentage of normal myelin (average values of sections) and final motor function scores were analyzed for the Pearson product-moment correlation coefficient. Results are presented as mean values±S.E. Values of p<0.05 were considered statistically significant.

## Supporting Information

Figure S1 **G-CSFR expression in naïve spinal cord. GFAP-positive astrocytes (A), MOSP-positive oligodendrocytes (B) and MAP-2-positive neurons (C) expressed G-CSFR.** Although the number of MOSP-positive oligodendrocytes was significantly different between the vehicle (D, open columns) and G-CSF groups (D, closed columns), all of the MOSP-positive oligodendrocytes expressed G-CSFR after SCI. Bar = 100 µm. Values are mean±S.E.M. *p<0.05.
(TIF)

Figure S2 **The number of MAP-2-positive neurons after SCI.** The number of MAP-2-positive neurons was significantly larger in the G-CSF group in the rostral and caudal segments (closed columns). Values are mean±S.E.M. *p<0.05.
(TIF)

Figure S3 **The influence of G-CSF on microglia\macrophages.** Double immunofluorescence study for ionized calcium-binding adaptor molecule 1 (Iba-1, as a marker for activated microglia and macrophages) and inducible nitric oxide synthase (iNOS, as a marker for Th1-driven activation of microglia/macrophages) or arginase-1 (a marker for Th2-driven activation of microglia/macrophages) was performed to elucidate G-CSF-mediated reaction and phenotypic alteration of macrophage/microglia. The number of Iba-1-positive cells in the G-CSF group was significantly smaller than that in the vehicle group in the rostral and caudal segments, whereas the ratios between iNOS (open columns) and arginase-1 (hatched or dotted columns) did not change in both the vehicle and G-CSF groups in lesioned spinal cord at any segments observed. Values are mean±S.E.M. *p<0.01.
(TIF)

Figure S4 **Apoptotic oligodendrocytes.** To further confirm the results of immunohistochemistry for apoptotic oligodendriocytes, we performed double immunofluorescence study for MOSP as another marker for oligodendrocytes and activated caspase-3 as a marker for apoptotic cells (A-C). The staining pattern was similar to that of the double fluorescence study for APC and activated caspase-3. Co-localization of MOSP and activated caspase-3 was further confirmed with orthogonal imaging obtained by laser confocal microscopy (D).
(TIF)
