# Supplementary figures and images for: Granulocyte Colony-Stimulating Factor (G-CSF) Protects Oligpdendrocyte and Promotes Hindlimb Functional Recovery after Spinal Cord Injury in Rats
Source: PLoS One. 2012 Nov 27;7(11):e50391. doi: 10.1371/journal.pone.0050391 (PMC3507692; doi:10.1371/journal.pone.0050391)

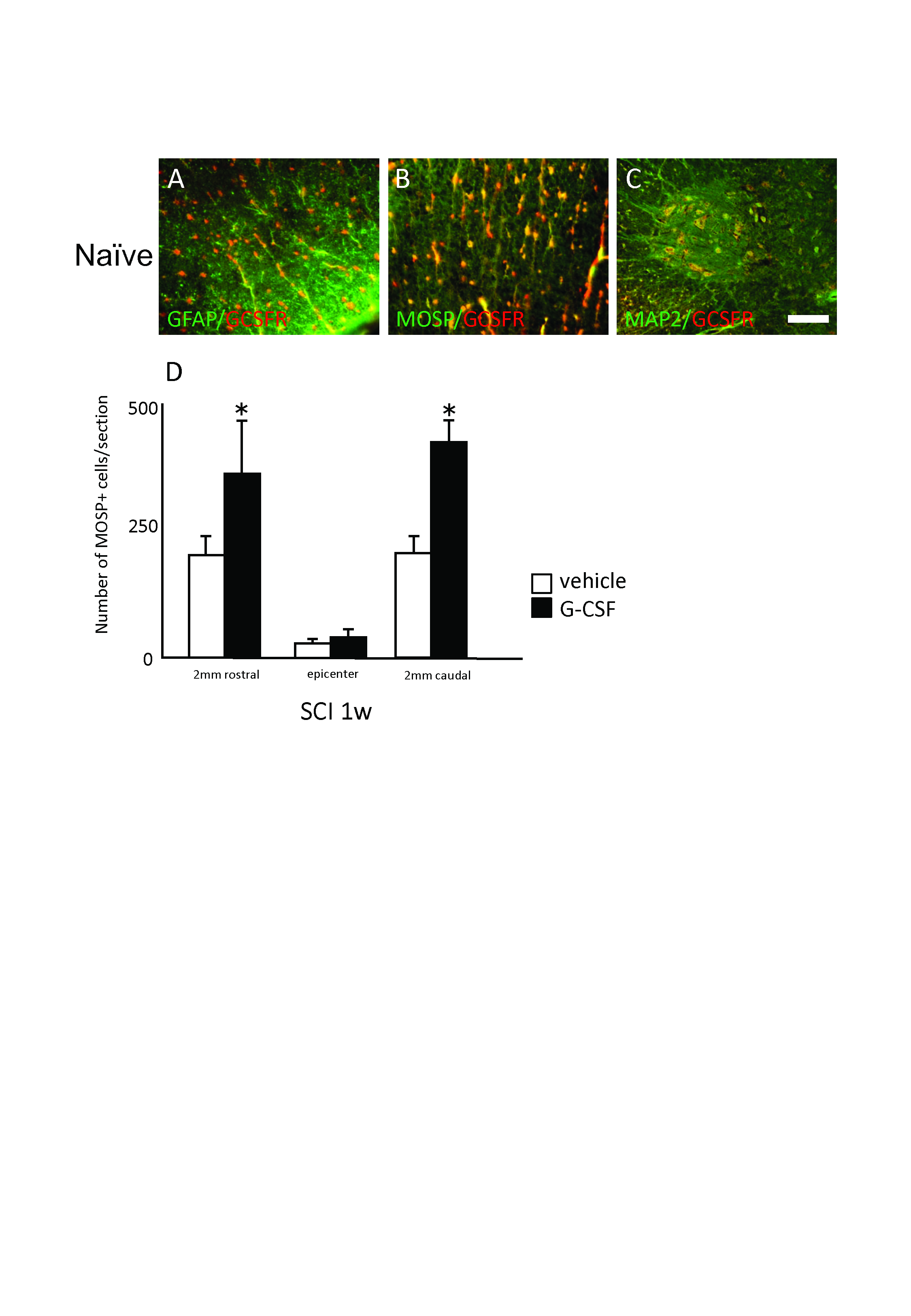

Supplement: Figure S1 — G-CSFR expression in naïve spinal cord. GFAP-positive astrocytes (A), MOSP-positive oligodendrocytes (B) and MAP-2-positive neurons (C) expressed G-CSFR. Although the number of MOSP-positive oligodendrocytes was significantly different between the vehicle (D, open columns) and G-CSF groups (D, closed columns), all of the MOSP-positive oligodendrocytes expressed G-CSFR after SCI. Bar = 100 µm. Values are mean±S.E.M. *p<0.05. (TIF) [file pone.0050391.s001.tif]

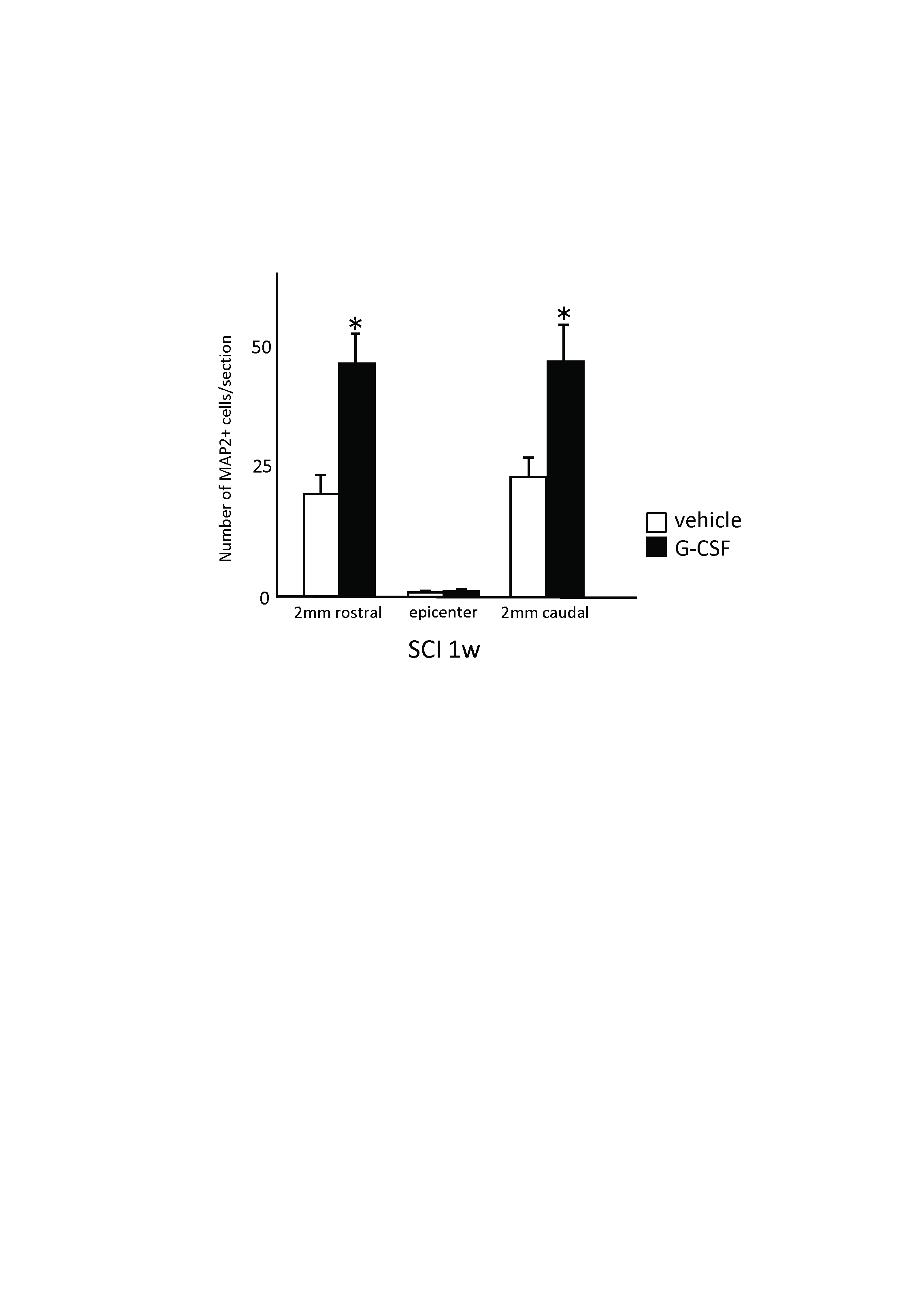

Supplement: Figure S2 — The number of MAP-2-positive neurons after SCI. The number of MAP-2-positive neurons was significantly larger in the G-CSF group in the rostral and caudal segments (closed columns). Values are mean±S.E.M. *p<0.05. (TIF) [file pone.0050391.s002.tif]

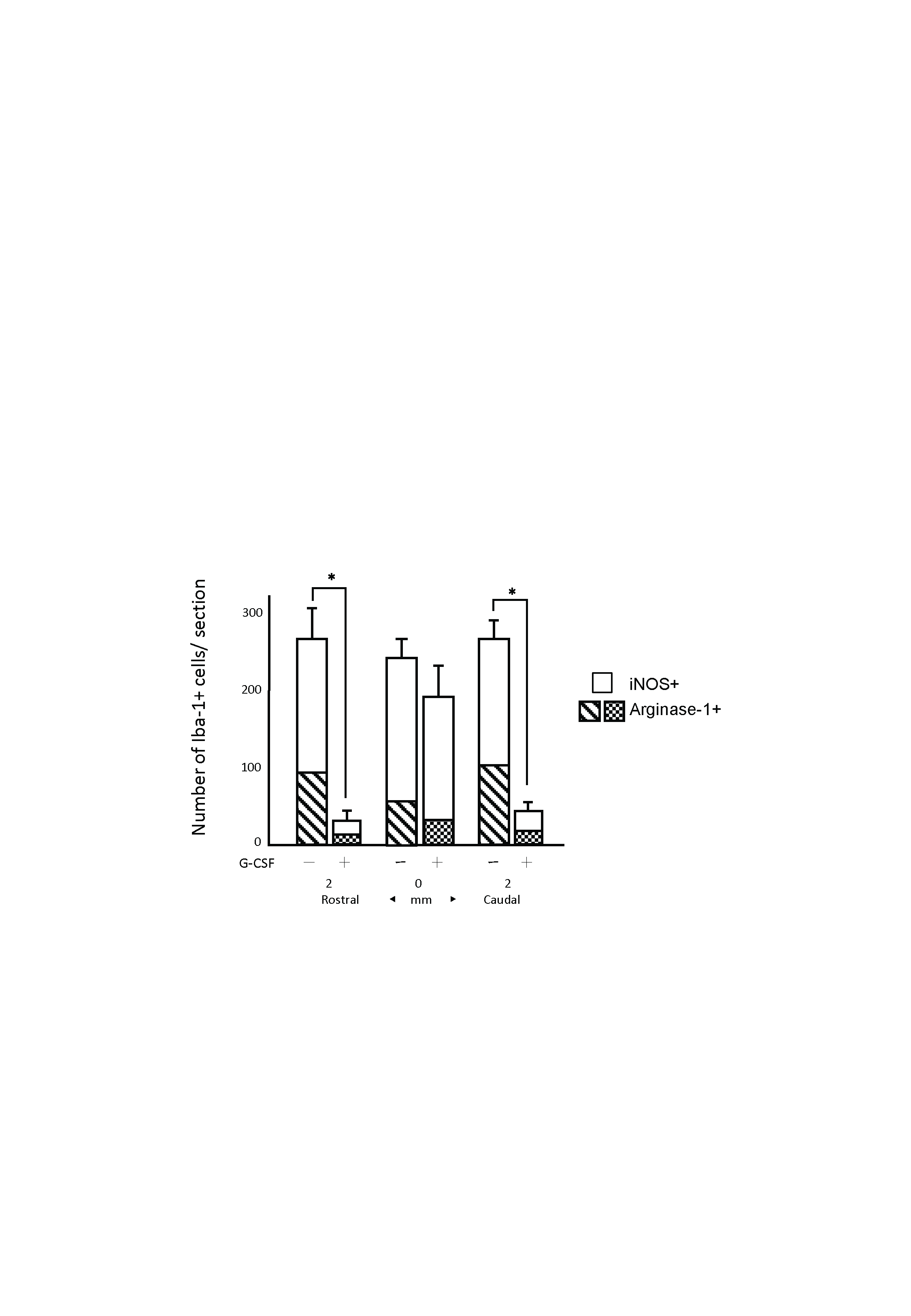

Supplement: Figure S3 — The influence of G-CSF on microglia\macrophages. Double immunofluorescence study for ionized calcium-binding adaptor molecule 1 (Iba-1, as a marker for activated microglia and macrophages) and inducible nitric oxide synthase (iNOS, as a marker for Th1-driven activation of microglia/macrophages) or arginase-1 (a marker for Th2-driven activation of microglia/macrophages) was performed to elucidate G-CSF-mediated reaction and phenotypic alteration of macrophage/microglia. The number of Iba-1-positive cells in the G-CSF group was significantly smaller than that in the vehicle group in the rostral and caudal segments, whereas the ratios between iNOS (open columns) and arginase-1 (hatched or dotted columns) did not change in both the vehicle and G-CSF groups in lesioned spinal cord at any segments observed. Values are mean±S.E.M. *p<0.01. (TIF) [file pone.0050391.s003.tif]

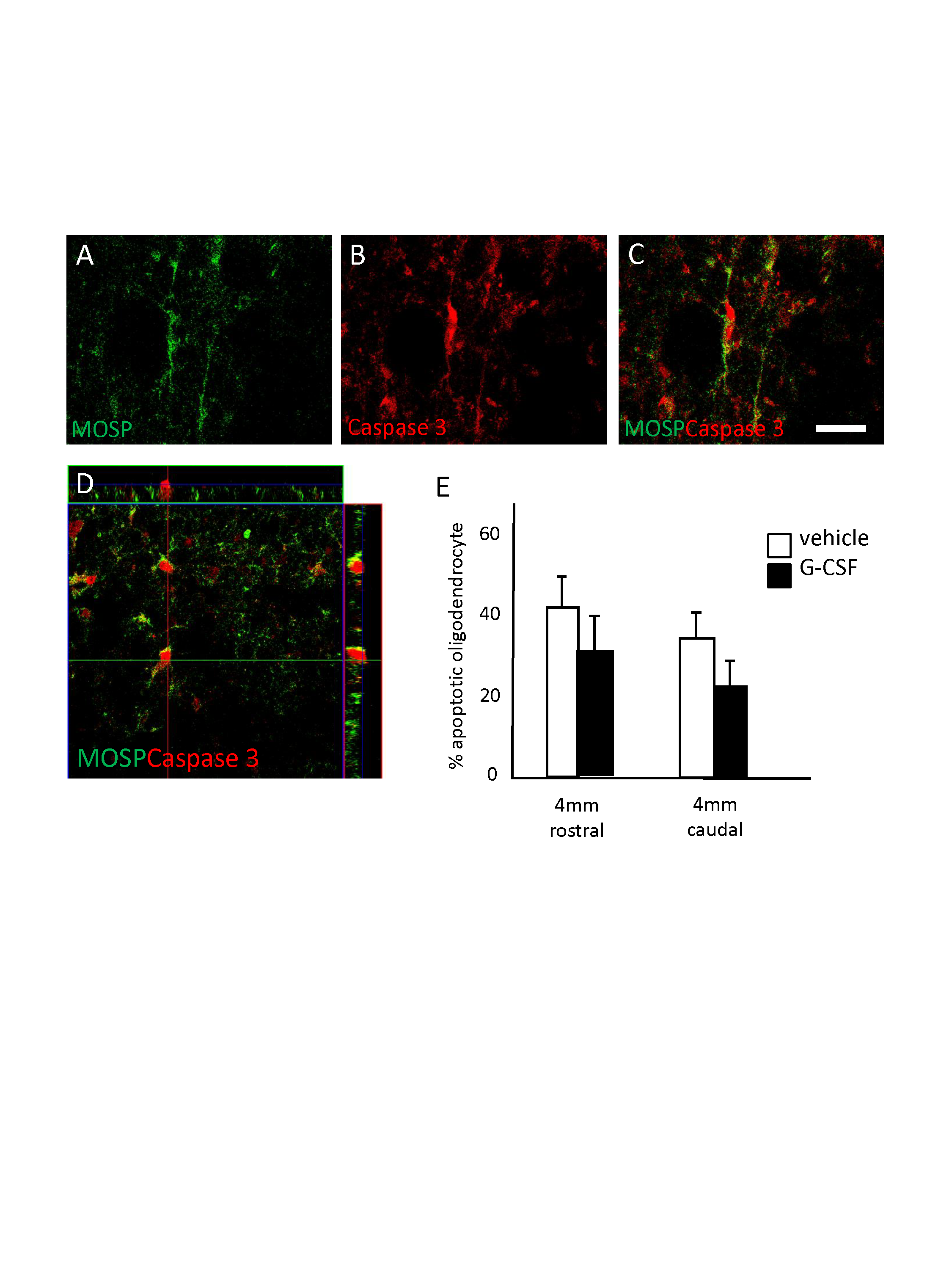

Supplement: Figure S4 — Apoptotic oligodendrocytes. To further confirm the results of immunohistochemistry for apoptotic oligodendriocytes, we performed double immunofluorescence study for MOSP as another marker for oligodendrocytes and activated caspase-3 as a marker for apoptotic cells (A-C). The staining pattern was similar to that of the double fluorescence study for APC and activated caspase-3. Co-localization of MOSP and activated caspase-3 was further confirmed with orthogonal imaging obtained by laser confocal microscopy (D). (TIF) [file pone.0050391.s004.tif]
